# Measuring and monitoring patient safety in hospitals in the Republic of Ireland

**DOI:** 10.1007/s11845-023-03336-3

**Published:** 2023-03-22

**Authors:** Yazeed Kaud, Darragh McKeon, Sinéad Lydon, Paul O’Connor

**Affiliations:** 1 Department of General Practice, School of Medicine, University of Galway, 1 Distillery Road, Newcastle, Co Galway H91 TK33 Galway, Ireland; 2 Department of Public Health, Saudi Electronic University, Riyadh, Saudi Arabia; 3 Health Services Executive, Dublin, Ireland; 4 Irish Centre for Applied Patient Safety and Simulation, University of Galway, Galway, Ireland; 5 School of Medicine, University of Galway, Galway, Ireland

**Keywords:** Hospital, Ireland, Measurement, Monitoring, Patient safety

## Abstract

**Background:**

Measuring and monitoring safety (MMS) is critical to the success of safety improvement efforts in healthcare. However, a major challenge to improving safety is the lack of high quality information to support performance evaluation.

**Aims:**

The aim of this study was to use Vincent et al.’s MMS framework to evaluate the methods used to MMS in Irish hospitals and make recommendations for improvement.

**Methods:**

The first phase of this qualitative study used document analysis to review national guidance on MMS in Ireland. The second phase consisted of semi-structured interviews with key stakeholders on their understanding of MMS. The MMS framework was used to classify the methods identified.

**Results:**

Six documents were included for analysis, and 24 semi-structured interviews were conducted with key stakeholders working in the Irish healthcare system. A total of 162 methods of MMS were identified, with one method of MMS addressing two dimensions. Of these MMS methods, 30 (18.4%) were concerned with past harm, 40 (24.5%) were concerned with the reliability of safety critical processes, 16 (9.8%) were concerned with sensitivity to operations, 28 (17.2%) were concerned with anticipation and preparedness, and 49 (30%) were concerned with integration and learning.

**Conclusions:**

There are a wide range of methods of MMS in Irish hospitals. It is suggested that there is a need to identify those methods of MMS that are particularly useful in reducing harm and supporting action and improvement and do not place a large burden on healthcare staff to either use or interpret.

**Supplementary Information:**

The online version contains supplementary material available at 10.1007/s11845-023-03336-3.

## Introduction

A commitment to safe healthcare is a policy goal of governments across the world. However, progress on delivering on this aspiration has been modest, with patients still suffering avoidable harm [1] and rates of harm remaining unchanged over time [2]. The impact that valid and reliable safety data can have on improvement is clear from many domains of healthcare [3]. Although safety data is complex and multi-faceted, it is vitally important to reducing patient harm [4]. This data is required to support the meaningful comparisons between the safety performance of different healthcare organisations and the assessment of the impact of safety interventions [1].

A recent review found that there has been relatively little research carried out on measuring and monitoring safety (MMS) in the healthcare system of the Republic of Ireland [5]. However, there is a recognition that MMS is central to patient safety improvement. This is evident in the Irish Health Service Executive (HSE) patient safety strategy which includes a commitment to ‘using information to improve safety’ (p.8) [6]. Recognising the complexity and multifaceted nature of the MMS in healthcare, Vincent et al. developed the MMS framework [4, 7]. The dimensions of the MMS framework are as follows:

1. Harm: has patient care been safe in the past? (e.g. national audits).
2. Reliability of safety critical processes: are our clinical systems and processes reliable? (e.g. monitoring of vital signs).
3. Sensitivity to operations: is care safe today? (e.g. safety walk-arounds).
4. Anticipation and preparedness: will care be safe in the future? (e.g. safety culture assessment).
5. Integration and learning: are we responding and improving? (e.g. aggregated analysis of incidents) [4, 7].

The framework has been used previously to categorise studies in systematic or scoping reviews of MMS in primary care [8], prehospital care [9], and secondary care in Ireland [5] and Saudi Arabia [10]. The study reported in this paper uses Vincent et al.’s [4, 7] MMS framework to classify the methods of MMS used within secondary care in Ireland. The aims of the study reported in this paper are to (1) examine how patient safety is measured and monitored in Irish hospitals; (2) map the methods of MMS in these hospitals onto the five dimensions of Vincent et al.’s [4, 7] MMS framework; and (3) reflect on the approaches used to MMS in Irish hospitals.

## Methods

### Research design

A qualitative descriptive approach was adopted for this study to: (1) support a document analysis of the guidance on MMS used in Irish hospitals; and (2) use semi-structured interviews to explore stakeholders’ perceptions about how patient safety is measured and monitored in Irish hospitals. This approach was based upon a study of methods of MMS use in Saudi Arabian hospitals [11].

### Phase one: document analysis

Methods of MMS in Irish hospitals that are described in national healthcare governance documents were identified and classified. Document analysis is a systematic method to review or evaluate documents [12]. The ‘ready materials, extract data, analyse data and distil (READ)’ approach [13] was utilised.

#### Inclusion criteria

The inclusion criteria required that documents were: national-level documents; explicitly discussed or described how patient safety is measured and monitored in Irish hospitals; produced by a national government agency/or an organisation affiliated with a national government agency; and written in English. No publication date or period was specified. Finally, in cases where a document had multiple versions, only the latest version of the document was included.

#### Exclusion criteria

Documents were excluded if: they did not describe how patient safety is measured and monitored in Irish hospitals; were not produced by an Irish government agency or an organisation affiliated with a national government agency; were focused on tracking performance progress (e.g. annual reports); were focused on the safety of one process only (e.g. medication safety); were focused on a particular method of measurement that is relevant to specific clinical practises (e.g. clinical audit); or were not written in English.

#### Search process

The search for relevant documents was completed in January 2022 and consisted of four steps intended to support the retrieval of government reports and policy documents:

1. An advanced google search was completed.
2. A search of the following electronic databases was conducted: Medline, CINAHL, OAIster, WHO IRIS, Lenus, and Google Scholar using various combinations of terms ‘measuring safety’, ‘monitoring safety’, and ‘measurement of safety’ (additional File 1 presents the search strategy used in complete detail).
3. Searches were conducted across the Irish Health Service Executive (HSE), Irish Department of Health, and Irish Health Information and Quality Authority (HIQA) websites using both their relevant search boxes and manual search.
4. Potential further related documents were identified through hand searching the reference lists of documents that met the inclusion criteria.

#### Document selection

The initial screening was completed by YK using the inclusion criteria to assess the potential for inclusion from the titles, abstracts, and/or executive summaries. Documents that appeared relevant were then downloaded for full-text review. A full-text review was completed by YK and POC to ensure the document met all items in the inclusion criteria. Decisions regarding the inclusion or exclusion of documents were agreed by consensus. All decisions were recorded in an Excel file.

#### Document analysis

YK and POC independently searched through each document and extracted all described methods of MMS. Only minor differences were found between the information extracted by the two reviewers. These differences were concerned with whether a particular method was one measure or could be split into two measures. Once the final list of MMS had been identified, YK, POC, and SL reviewed each measure and reached a decision by consensus as to which dimension of Vincent et al.’s [4, 7] MMS framework it addressed.

### Phase two: semi-structured interviews with key stakeholders

The aim of the second phase of the study was to explore what key stakeholders know about how safety is measured and monitored in the Irish healthcare system.

#### Ethical review

The study was approved by the Clinical Research Ethics Committee, Galway University Hospitals (Ref: C.A.2604). All participants provided signed written informed consent.

#### Sampling and recruitment of participants

Participants for the interviews were drawn from three different stakeholder groups: (1) policy makers; (2) medical doctors; and (3) nurses. Recruitment of participants was through a combination of purposive and snowball sampling.

#### Development of interview guide

The semi-structured interview guide that was used is shown in Table 1. The design of the interview guide was derived from the five dimensions of Vincent et al.’s [4, 7] MMS framework. The interview questions were prepared in accordance with best practises for the formulation of interview questions [14, 15]. The interviews were conducted using an interview schedule developed to obtain information about perceptions of MMS in Saudi Arabian hospitals [11].

**Table 1:** Interview guide used to engage participants in discussion around measuring and monitoring safety in Ireland

1. In the Irish healthcare system, how is harm to patients measured and monitored?
1.1. What are the strengths and limitation of methods used?
1.2. Are there other methods of measuring and monitoring harm that you think should be used? and if so, what are these and why do you think they’d be useful?
2. What methods are in place to assess whether our clinical systems, processes and behaviour reliable?
2.1. What are the strengths and limitation of each of these methods?
2.2. Are there other methods of measuring and monitoring standardised clinical practice that you think should be used?
3. What methods are in place to assess whether care is safe in hospitals in Ireland today?
3.1. What are the strengths and limitation of each of these methods?
3.2. Are there other methods of measuring and monitoring whether patient care is safe today you think should be used? and if so, what are these and why do you think they be useful?
4. What methods are in place to anticipate and reduce future risks to patients’ hospitals in Ireland?
4.1. What are the strengths and limitation of each of these methods?
4.2. Are there other methods of improving the anticipation and reduction of future risk to patients that you think should be used? and if so, what are these and why do you think they be useful?
5. What methods are in place to promote learning from issues and improving the level of patient safety in hospitals in Ireland?
5.1.What are the strengths and limitation of each of these methods?
5.2. Are there other methods of prompting learning that you think should be used, and if so, what are these and why do you think they be useful?

#### Procedure

All interviews were carried out from January 2022 to June 2022. After receiving written informed consent, YK and DM conducted the interviews via video conference call. The audios of the calls were recorded.

#### Interview analysis

The purpose of the interview analysis was to identify the methods of MMS which the participants knew were being used in Irish hospitals. These methods of MMS were categorised using Vincent et al.’s [4, 7] MMS framework. The methods of MMS described by the interviewees were extracted from the transcripts by YK and DM and then reviewed by POC. Decisions on categorisation were made by consensus.

## Results

### Phase one: document analysis

**Fig. 1:**
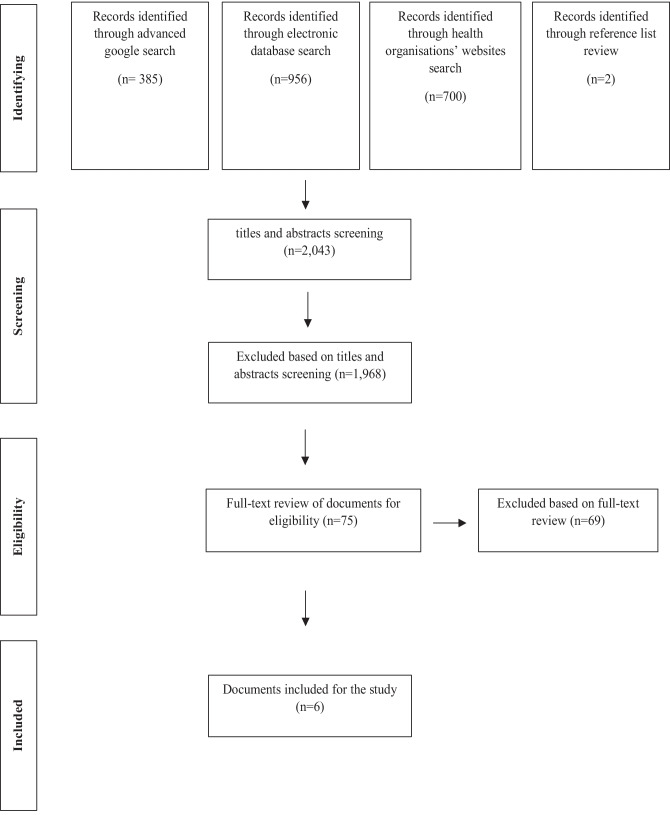
Flow diagram depicting study selection for document analysis

A total of six documents were found to meet the inclusion criteria. All six documents had been published since 2008. The search process for identifying documents that met the inclusion criteria is shown in Fig. 1. A summary overview of these documents is provided in Table 2.

**Table 2:** A summary of key information of each included document

Title of the document	Year published	Pages	Prepared by	Stated aim	Target population	Setting	Number of methods of MMS measures included
Patient Safety Strategy 2019–2024	2019	26	Health Service Executive (HSE)	To improve the safety of all patients by identifying and reducing preventable harm within the health and social care system	‘Patient’ refers to all people who attend/use health and social care services. “Staff” includes all healthcare professionals (HCPs), clinicians, support workers, managers, and administration	Every level of health and social care services, within both community and acute hospital services	*N* = 52*
							1. Harm, 12 (22.6%)
							2. Reliability of safety critical processes, 3 (5.6%)
							3. Sensitivity to operations, 10 (18.8%)
							4. Anticipation and preparedness, 12 (22.6%)
							5. Integration and learning, 16 (30.2%)
Building a Culture of Patient Safety: Report of the Commission on Patient Safety and Quality Assurance	2008	227	Government of Ireland. The Commission on Patient Safety and Quality Assurance	To provide recommendations for a framework of patient safety and quality	‘Patient’ refers to all people who use health and social care services. ‘Clinician’ refers to all HCPs involved in clinical work	All levels of the health system	*N* = 6
							1. Harm, 1 (16.6%)
							2. Reliability of safety critical processes, 1 (16.6%)
							3. Sensitivity to operations, 3 (50%)
							4. Anticipation and preparedness, 0 (%)
							5. Integration and learning, 1 (16.6%)
Acute Hospitals Key Performance Indicator Metadata 2021	2021	118	Health Service Executive (HSE)	Key Performance Indicator (KPI) metadata templates are completed for all National Service Plan metrics and provide the most up-to-date information relating to KPIs	Information includes definition, rationale, reporting frequency, and data source. They underpin data quality, accessibility, and records management for data collectors and inform users of data	Acute hospitals	*N* = 15
							1. Harm, 5 (33.3%)
							2. Reliability of safety critical processes, 10 (66.6%)
							3. Sensitivity to operations, 0 (%)
							4. Anticipation and preparedness, 0 (%)
							5. Integration and learning, 0 (%)
National Standards for the Conduct of Reviews of Patient Safety Incidents 2017	2017	60	Health Information and Quality Authority (HIQA) and Mental Health Commission (MHC)	To promote a framework for best practice in the conduct of reviews of patient safety incidents in order to set a standard for cohesive, person-centred reviews of such incidents	The standards were developed with an initial focus on services-specific for acute hospitals and mental health services	Acute hospitals under HIQA’s remit and mental health services under the remit of the MHC	*N* = 37
							1. Harm, 2 (5.4%)
							2. Reliability of safety critical processes, 16 (43.2%)
							3. Sensitivity to operations, 3 (8.2%)
							4. Anticipation and preparedness, 5 (13.5%)
							5. Integration and learning, 11 (29.7%)
Incident Management Framework 2020	2020	42	Health Service Executive (HSE)	To provide an overarching practical approach, based on best practice, to assist providers of HSE and HSE-funded services to manage all incidents (clinical and non-clinical) in a manner that is cognisant of the needs of those affected and supports services to learn and improve	Staff, managers, and Senior Accountable Officer (SAO) and related teams/committees in HSE and HSE-funded agencies	All publicly funded health and social care services provided in Ireland	*N* = 23
							1. Harm, 6 (26%)
							2. Reliability of safety critical processes, 5 (21.7%)
							3. Sensitivity to operations, 0 (%)
							4. Anticipation and preparedness, 1 (4.3%)
							5. Integration and learning, 11 (47.8%)
National Standards for Safer Better Healthcare	2012	157	Health Information and Quality Authority (HIQA)	The National Standards for Safer Better Healthcare aim to give a shared voice to the expectations of the public, service users, and service providers. They also provide a roadmap for improving the quality, safety, and reliability of healthcare	Service users and service providers. The term service provider refers to any person, organisation, or part of an organisation delivering healthcare services, as described in the Health Act 2007	These National Standards apply to all healthcare services (excluding mental health) provided or funded by the HSE including, but not limited to hospital care, ambulance services, community care, primary care, and general practice	*N* = 29
							1. Harm, 4 (13.7%)
							2. Reliability of safety critical processes, 5 (17.2%)
							3. Sensitivity to operations, 0 (%)
							4. Anticipation and preparedness, 10 (34.4%)
							5. Integration and learning, 10 (34.4%)

^*^One of the methods of MMS was classified under two dimensions, and so these percentages are calculated using a denominator of 53

*HCPs* healthcare professionals

A total of 162 methods of MMS were identified across the six documents (see Table 2 and Additional File 2 for a list of these methods and how they were classified). Of these MMS methods, 30 (18.4%) were concerned with past harm, 40 (24.5%) were concerned with the reliability of safety critical processes, 16 (9.8%) were concerned with sensitivity to operations, 28 (17.2%) were concerned with anticipation and preparedness, and 49 (30%) were concerned with integration and learning. One method of MMS addressed two of the safety dimensions (past harm and integration and learning); therefore, the percentages are calculated out of 163.

### Phase two: semi-structured interviews with key stakeholders

The mean duration of the interviews was 25 min (SD = 11 min 58 s). The 24 participants included 18 frontline healthcare workers (nine doctors and nine nurses) and six healthcare policy makers. Of the 24 participants, 14 were women and 10 were men. The participants reported a mean of 13 years of professional experience (range = 3–31 years). Twenty-one (87.5%) of the participants worked in teaching hospitals, and three (12.5%) in national health regulation organisations.

The MMS methods reported by interviewees are shown in Table 3. Illustrative quotes pertaining to the most commonly reported measures in each safety domain are provided in Table 4. The interviewees described a total of 76 methods of MMS. Of these methods of MMS, 14 (18.4%) were concerned with past harm, 25 (33%) were concerned with the reliability of safety critical processes, 8 (10.5%) were concerned with sensitivity to operations, 15 (19.7%) were concerned with anticipation and preparedness, and 14 (18.4%) were concerned with integration and learning.

**Table 3:** Methods reported by participants to measure and monitor patient safety in Irish hospitals

Dimension	No	Reported methods of measuring and monitoring safety	Number of participants reported the measure (no.) %		
			(18) 87.5% Front-line healthcare staff	(6) 12.5% Policy makers	(24) 100% All
1. Harm	1.	Incident reporting systems	(15) 83.3%	(4) 66.6%	(19) 79.2%
	2.	National Incident Management System (NIMS)	-	(1) 16.6%	(1) 4.2%
	3.	Hospital-acquired complications	-	(1) 16.6%	(1) 4.2%
	4.	Hospital In-Patient Enquiry (HIPE)	-	(1) 16.6%	(1) 4.2%
	5.	Mortality and morbidity rates	(5) 27.8%	-	(5) 20.8%
	6.	Patient safety indicators	(1) 5.5%	-	(1) 4.2%
	7.	Incidence of falls	(2) 11%	-	(2) 8.3%
	8.	Pressure ulcer rates	(3) 16.7%	-	(3) 12.5%
	9.	State Claims Agency	(1) 5.5%	-	(1) 4.2%
	10.	Medication error reporting	(3) 16.7%	-	(3) 12.5%
	11.	Rates of healthcare-associated infections (HCAIs)	(1) 5.5%	(1) 16.6%	(2) 8.3%
	12.	Readmission rates	(1) 5.5%	-	(1) 4.2%
	13.	Patient satisfaction surveys	-	(1) 16.6%	(1) 4.2%
	14.	Patients’ complaint systems	(1) 5.5%	-	(1) 4.2%
2. Reliability of safety critical processes	1.	Monitoring compliance to hand hygiene	(1) 5.5%	(2) 33.3%	(3) 12.5%
	2.	Observation of safety critical behaviours	(2) 11%	-	(2) 8.3%
	3.	Monitoring national standards	(5) 27.8%	(1) 16.6%	(6) 25%
	4.	National/international accreditation	(1) 5.5%	-	(1) 4.2%
	5.	Inspections to monitor compliance against standards and guideline	(4) 22.2%	(1) 16.6%	(5) 20.8%
	6.	Venous thromboembolism risk assessment	(1) 5.5%	-	(1) 4.2%
	7.	Key performance indicators of patient safety goals	(3) 16.7%	(1) 16.6%	(4) 16.7%
	8.	Audit of equipment	(6) 33.3%	-	(6) 25%
	9.	Infection control checklists	(1) 5.5%	-	(1) 4.2%
	10.	Clinical audit	(14) 77.8%	(4) 66.6%	(18) 75%
	11.	Patient observation charts	(4) 22.2%	-	(4) 16.7%
	12.	Double checks by other staff members	(7) 38.9%	-	(7) 29.2%
	13.	Monitoring of vital signs	(1) 5.5%	-	(1) 4.2%
	14.	Quality and safety monthly governance meeting	-	(1) 16.6%	(1) 4.2%
	15.	Patient administration systems	-	(1) 16.6%	(1) 4.2%
	16.	Specialty-specific data management systems	-	(1) 16.6%	(1) 4.2%
	17.	Turnaround times (TAT)	-	(1) 16.6%	(1) 4.2%
	18.	Early warning score	(6) 33.3%	(2) 33.3%	(8) 33.3%
	19.	Armbands to identify patients at risk	(1) 5.5%	-	(1) 4.2%
	20.	Surgical checklist	(3) 16.7%	-	(3) 12.5%
	21.	Systems to check bed availability	(1) 5.5%	-	(1) 4.2%
	22.	Preoperative assessment clinic	(1) 5.5%	-	(1) 4.2%
	23.	Medication administration checklists	(1) 5.5%	-	(1) 4.2%
	24.	Staff assessment and credentialling	(1) 5.5%	-	(1) 4.2%
	25.	Monitoring delays in treatment	(1) 5.5%	-	(1) 4.2%
3. Sensitivity to operations	1.	Safety walk-arounds	(5) 27.8%	(1) 16.6%	(6) 25%
	2.	Talking to patients	(3) 16.7%	-	(3) 12.5%
	3.	Safety huddles	(4) 22.2%	(2) 33.3%	(6) 25%
	4.	Briefings and debriefings	(2) 11%	-	(2) 8.3%
	5.	Observation and conversations with clinical teams	(7) 38.9%	(1) 16.6%	(8) 33.3%
	6.	Ward rounds and routine reviews of patients and working conditions	(2) 11%	-	(2) 8.3%
	7.	Handover and handouts	(4) 22.2%	-	(4) 16.7%
	8.	Real-time monitoring and feedback in anaesthesia	(1) 5.5%	-	(1) 4.2%
4. Anticipation and preparedness	1.	Failure mode and effect analysis (FMEA) to identify risks	(1) 5.5%	-	(1) 4.2%
	2.	Staff assessment and credentialing	(3) 16.7%	-	(3) 12.5%
	3.	Risk registers	-	(4) 66.6%	(4) 16.7%
	4.	Anticipated staffing levels and skill mix	(7) 38.9%	-	(7) 29.2%
	5.	Screening for embolism	(1) 5.5%	-	(1) 4.2%
	6.	Timely safety alerts	-	(1) 16.6%	(1) 4.2%
	7.	Comprehensive hazard identification risk assessment	-	(1) 16.6%	(1) 4.2%
	8.	A hospital emergency management plan that is aligned with the city’s emergency management plan	-	(1) 16.6%	(1) 4.2%
	9.	Comprehensive risk assessments of patient at admission	(4) 22.2%	-	(4) 16.7%
	10.	Fall risk assessment	(1) 5.5%	-	(1) 4.2%
	11.	Waterlow skin assessment	(2) 11%	-	(2) 8.3%
	12.	Malnutrition Universal Screening Tool (MUST)	(2) 11%	-	(2) 8.3%
	13.	Nursing pools	(1) 5.5%	-	(1) 4.2%
	14.	Risk prediction scores in anaesthesia	(1) 5.5%	-	(1) 4.2%
	15.	Preoperative assessment of patients	(2) 11%	-	(2) 8.3%
5. Integration and learning	1.	Analysis of incidents and feedback leading to the implementation of safety lessons	(8) 44.4%	(4) 66.6%	(12) 50%
	2.	Learning from audits	(1) 5.5%	-	(1) 4.2%
	3.	Learning from patient safety alerts	-	(1) 16.6%	(1) 4.2%
	4.	Learning from patients’ complaints	(2) 11%	(1) 16.6%	(3) 12.5%
	5.	Learning from meetings and discussion of sentinel events	(2) 11%	-	(2) 8.3%
	6.	Debriefing sessions to provide feedback on clinical performance	(3) 16.7%	-	(3) 12.5%
	7.	Learning from root cause analysis	(2) 11%	(1) 16.6%	(3) 12.5%
	8.	Learning from excellence	-	(1) 16.6%	(1) 4.2%
	9.	Learning reported in research papers from other health organisations	-	(2) 33.3%	(2) 8.3%
	10.	learning from safety networks that involve local and national health agencies	(1) 5.5%	(2) 33.3%	(3) 12.5%
	11.	After action reviews (AAR)	-	(1) 16.6%	(1) 4.2%
	12.	Learning from international experience reported in the literature	-	(1) 16.6%	(1) 4.2%
	13.	Simulation sessions following patient safety incidents	(5) 27.8%	-	(5) 20.8%
	14.	Learning from mortality and morbidity reviews	(2) 11%	-	(2) 8.3%

**Table 4:** Example quotes from the interview transcripts

**Dimension**	**Example quotes**
1. **Harm**	Incident reports
	‘There is the Q-Pulse system, which is a self-reporting system in the hospital, and there are different categories for reporting, things related to work and things related to safety, and other related to other things to improve quality and safety and so on’ (Doctor 1)
	‘I suppose the most prominent method would be the use of incident report systems’ (Doctor 6)
	‘Within Irish hospitals, the method used to measure, and monitor harm would mainly be incident reporting systems’ (Policy maker 4)
	Mortality and morbidity rates
	“One major thing would be our departments use of morbidity and mortality rates” (Doctor 6)
2. **Reliability of safety critical processes**	Clinical audit
	‘Clinical audit, we have a very robust audit and quality improvement department in the hospital, doctors and nurses are invited to carry out audits’ (Doctor 6)
	Early warning score
	“The early warning score, that was another initiative that was brought in that's countrywide as well” (Nurse 7)
3. **Sensitivity to operations**	Observation and conversations with clinical teams
	‘Direct observation of procedures, whereby a senior will initially observe you performing procedure and in a structured manner and observe the different steps and analyse what you’re doing and then deliver feedback afterwards’ (Doctor 6)
	Safety walk-arounds
	‘There are ground round or ward round. I think every senior in any team should be able to do at least one round every day with the juniors, and there should be a bigger round done every week for example in presence of all seniors’ (Doctor 2)
	Safety huddles
	‘There is also what we call the safety pause or the safety huddle, so during the safety huddles, we’ll discuss whether there has been anything wrong, or there is something that isn’t working properly, and needs to be fixed that is related to patient safety, we also discuss whether there are any new guidelines or protocols’ (Nurse 2)
4. **Anticipation and preparedness**	Anticipated staffing levels and skill mix
	‘anticipating staffing levels, for example, during the winter, there’s going to be a rise in your flu cases, so anticipating that we’re going to need more staff nurses at that time’ (Nurse 9)
	Comprehensive risk assessments of patients at admission
	‘There are assessments as soon as the admission takes place in order for us to avoid harm. We will assess several factors, and then we know, does this patient need more care?’ (Nurse 3)
5. **Integration and learning**	Analysis of incidents and feedback leading to the implementation of safety lessons
	‘When an incident report is filed, this is discussed by specific team that manages incident reports and usually they discuss it with the person that’s involved, not in terms of putting blame, but in terms of addressing how the mistake happened and how to prevent it’ (Doctor 5)
	Simulation sessions following patient safety incidents
	‘So, I think simulation plays a big role. We certainly do that. From time to time will run multi-disciplinary simulations with the nurses and sometimes with ICU or other teams’ (Doctor 8)

The most frequently reported MMS method for past harm was incident reporting (mentioned by 19; 79.2% of the interviewees). In addition, incident reports were by far the most commonly reported method of MMS across all dimensions. Clinical audit was the most frequently mentioned MMS method for the reliability of safety critical process dimension (mentioned by 18; 75% of the interviewees). Observation and conversations with clinical teams were the most frequently described MMS method for the sensitivity to operation dimension (mentioned by 8; 33.3% of the interviewees), followed by safety walk-arounds (mentioned by 6; 25% of the interviewees) and safety huddles (which were also mentioned by 6; 25% of the interviewees). Analysis of incidents and feedback leading to the implementation of safety lessons (mentioned by 12; 50% of interviewees) was the most often reported MMS method in the dimension of integration and learning.

## Discussion

A fundamental challenge to improving patient safety in healthcare is a dearth of high-quality information that allows organisations and individual practitioners to analyse their performance, define priorities, and identify areas of deficiency and risk. In this study, we examined how patient safety is measured and monitored in Irish hospitals, mapped these methods onto Vincent et al.’s [4, 7] MMS framework, and reflected on the meaning of these findings for MMS in Irish hospitals.

Considering both the findings from the document analysis and interviews, it can be seen that a wide variety of methods are used to MMS in Irish hospitals. However, although there were measures from across all five of the MMS framework dimensions, there was some variability in the number of methods within the dimensions. Measures of the reliability of safety critical processes were the most commonly identified methods of MMS in the document analysis and interviews. This may reflect the amount of routine safety data that are collected in Irish hospitals. The dimension with the smallest number of measures was sensitivity to operations. A possible explanation for this finding is that these measures tend to be qualitative (e.g. talking with patients and staff, observing staff) [16]. Such ‘soft intelligence’ is generally more difficult to collect, and analyse, than is the case for quantitative data [17]. However, this qualitative data can provide valuable insights into issues that may not be possible to gain from quantitative methods of MMS. Automated language analysis methods are beginning to be used to analyse qualitative patient safety data [18]. Therefore, there is the potential for the analysis of qualitative data to become much easier, and faster, than in the past.

Of all the measures identified by the interviewees across the five MMS safety dimensions, incident reports were the most common method of MMS—identified by almost four-fifths of the interviewees. However, the international literature suggests that healthcare organisations may overly rely on the analysis of past events and past harms as a source of safety performance information [19–21]. Thus, whilst it is positive that frontline healthcare workers are aware of the importance of collecting information on adverse events, it is important that the limitations of this particular measure are recognised. It is well known that reporting systems underestimate the prevalence of patient safety incidents [22] and overestimate the severity of harm [23]. Therefore, it is important to avoid an over-reliance on incident reports and past harm more generally, as the primary source of safety data.

The second most common method of MMS identified by the interviewees was clinical audit—identified by three quarters of the interviewees. Clinical audits are widely used in many healthcare systems to assess clinical performance against pre-set standards and use the data to improve practise [24]. Nevertheless, despite their widespread use, audits’ effectiveness as a practise-improvement strategy is often presumed, rather than supported by robust evidence [24]. A review of the impact of clinical audit on healthcare workers practise and patient outcomes concluded that audits resulted in a little change, only 4% of the studies which used clinical audit resulted in an increase in the desired practise [25]. Research found that audits were more likely to be successful when there was low baseline performance, feedback was offered several times by a colleague or supervisor in both verbal and written formats, and defined objectives and an action plan were included [24]. Moreover, clinical audit places a considerable burden on staff to complete [26]. It is certainly not suggested that the health service abandons the practise of clinical audit. However, there is a need to consider how to reduce the resources, and burden, of MMS [3].

### Recommendations

Our study has shown that there are methods of MMS from across all five dimensions of the framework. However, despite collecting large volume of safety data about hospital care, it still remains challenging to determine the safety of the delivery of care [4, 7]. It has been suggested that healthcare stakeholders could get the information they need with a quarter of what is currently being spent on MMS [27]. The WHO has also identified the burden of collecting and analysing data as a barrier to MMS [3]. Therefore, to improve patient safety in the Irish healthcare system, we recommend a number of issues that should be addressed.

- **Reliability of safety data**. The reliability of most safety data is unknown, and in some cases the reliability may actually be known to be problematic (e.g. hand hygiene compliance). If measures are poorly designed, this can lead to ‘gaming’, where targets are achieved but the intended changes in practise are not [28]. Therefore, consideration needs to be given to identifying which methods of MMS result in reliable data.
- **Fragmentation of data**. There are a huge range of methods of MMS focused at different levels of a healthcare organisation (e.g. units, hospitals), by different organisations (e.g. HIQA, Department of Health). This fragmentation of data creates challenges for healthcare professionals and managers in identifying where improvement efforts should be made, and whether these efforts are effective [1]. It is recommended that there is a consolidation of efforts across these agencies to avoid repetition and overlap of efforts.
- **Quantity of safety data.** A total of 162 methods of MMS were identified from the document analysis, and 76 methods of MMS identified from the interviews. This quantity of data can be overwhelming for healthcare workers and managers. There is a need for safety data to be readily interpreted so that safety issues can be identified at unit, hospital, and national levels. Measures that are too burdensome or lack credibility may alienate clinicians and lead to confusion about the impact of interventions [29]. It is suggested that the perspectives of all stakeholders in healthcare should be taken to identify key measures, from across the five MMS domains, that are particularly useful in supporting action and improvement, and do not place a large burden on healthcare staff to use.
- **Lack of ownership of the data.** Much of the data is focused on measures generated externally to a clinical team, so the teams may not perceive the data as being related to their performance [30]. Consideration should be given to how to engage front-line clinical staff in MMS so that they feel some ownership and are empowered to act upon the data, and involving them, and other stakeholders, in identifying meaningful methods of MMS.

### Limitations

There are a number of limitations of this research that should be acknowledged. The main limitation is that this paper only focused on MMS in the Irish healthcare system. However, the findings are similar to those derived from a study that utilised the same methodology to consider the MMS in the Saudi Arabian healthcare system [11]. Therefore, there would appear to be generalisability of the findings to other healthcare systems. As is the case with other qualitative approaches, our study could be critiqued due to the subjectivity of this type of research. However, these issues were mitigated through the rigorous approach we took to the data collection and analysis. The focus of the interviews was on identifying the methods of MMS, and did not include an analysis of the quality of the data collected using these approaches. Finally, we only considered national-level publications in the document analysis, which may have led to the exclusion of useful hospital-level documents. However, the difficulty in systematically accessing hospital-level documents was a barrier to their inclusion.

## Conclusion

There are a wide range of methods of MMS in Irish hospitals. However, having larger numbers of methods of MMS does not necessarily correspond to a robust safety surveillance system. It is suggested that the input of all stakeholders in healthcare are gathered to identify particularly key measures, across the five MMS domains, in order to identify those methods of MMS that are particularly useful in reducing harm, supporting action and improvement, and do not place a large burden on healthcare staff to use or interpret.

### Supplementary Information

Below is the link to the electronic supplementary material.

Supplementary file1 (DOCX 32 KB)

Supplementary file2 (DOCX 33 KB)

Supplementary file3 (DOCX 27 KB)

Supplementary file4 (DOCX 70 KB)

## Data Availability

All data arising from the document analysis is presented either in the article or within the included supplemental material. Interview data is summarised within the article. The ethical approval for this study does not include provision for sharing interview transcripts.
