# Supplementary material for: Measuring and monitoring patient safety in hospitals in the Republic of Ireland
Source: Ir J Med Sci. 2023 Mar 22;192(6):2581–93. doi: 10.1007/s11845-023-03336-3 (PMC10692269; doi:10.1007/s11845-023-03336-3)
Supplement: Supplementary file 3 — Supplementary file3 (DOCX 27 KB) [file 11845_2023_3336_MOESM3_ESM.docx]

**Additional file 1 (Search strategy)**

# **Advanced google search**

| **#** | **Search terms** | | **Total search yield** | **Potentially relevant records / full text review** | **Included** |
| --- | --- | --- | --- | --- | --- |
| 1 | “Measuring safety” | AND "Ireland" | n=94 | n=9 |  |
| 2 |  | AND "Irish hospitals" | n=77 | n=9 |  |
| 3 |  | AND "Irish healthcare" | n=79 | n=3 |  |
| 4 | “Monitoring safety” | AND "Ireland" | n=90 | n=3 |  |
| 5 |  | AND "Irish hospitals" | n=4 | n=0 |  |
| 6 |  | AND "Irish healthcare" | n=7 | n=0 |  |
| 7 | “Measurement of safety” | AND "Ireland" | n=19 | n=0 |  |
| 8 |  | AND "Irish hospitals" | n=5 | n=1 |  |
| 9 |  | AND "Irish healthcare" | n=10 | n=0 |  |
| Total= | | | N=385 | N=25 | N=5 |

# **MEDLINE**

| **Search strategy** | **Total search yield** | **Potentially relevant records / full text review** | **Included** |
| --- | --- | --- | --- |
| 1. exp patient safety/  2. safe*2.ti,ab.  3. exp medical errors/  4. (medica* adj1 error*1).ti,ab.  5. (adverse adj1 event*1).ti,ab.  6. (sentinel adj1 event*1).ti,ab.  7. (patient adj1 safety adj1 incident*1).ti,ab.  8. (patient adj1 harm*).ti,ab.  9. (healthcare adj1 quality).ti,ab.  10. (quality adj1 care).ti,ab.  11. 1 or 2 or 3 or 4 or 5 or 6 or 7 or 8 or 9 or 10  12. measur*.ti,ab.  13. monitor*.ti,ab.  14. assess*.ti,ab.  15. evaluat*.ti,ab.  16. 12 or 13 or 14 or 15  17. exp hospital/  18. hospital*1.ti,ab.  19. exp secondary care/  20. (secondary adj1 care).ti,ab.  21. exp tertiary healthcare/  22. 17 or 18 or 19 or 20 or 21  23. exp ireland/  24. irish.ti,ab.  25. 23 or 24  26. 11 and 16 and 22 and 25 | 226 | 2 | 0 |

# **CINAHL**

| **Search strategy** | **Total search yield** | **Potentially relevant records / full text review** | **Included** |
| --- | --- | --- | --- |
| S27 S12 AND S18 AND S22 AND S26  S26 S23 OR S24  S24 TI irish OR AB irish  S23 TI ireland OR AB ireland  S22 S19 OR S20 OR S21  S21 TI "tertiary care" OR AB "tertiary care"  S20 TI "secondary care" OR AB "secondary care"  S19 TI hospital* OR AB hospital*  S18 S13 OR S14 OR S15 OR S16 OR S17  S17 TI evaluat* OR AB evaluat*  S16 TI assess* OR AB assess*  S15 TI manag* OR AB manag*  S14 TI monitor* OR AB monitor*  S13 TI measur* OR AB measur*  S12 S1 OR S2 OR S3 OR S4 OR S5 OR S6 OR S7 OR S8 OR S9 OR S10 OR S11  S11 TI "quality *care" OR AB "quality *care"  S10 TI "healthcare quality" OR AB "healthcare quality"  S9 TI "iatrogenic disease" OR AB "iatrogenic disease"  S8 TI "patient harm" OR AB "patient harm"  S7 TI "patient safety incident" OR AB "patient safety incident"  S6 TI "sentinel event" OR AB "sentinel event"  S5 TI "adverse event" OR AB "adverse event"  S4 TI "risk management" OR AB "risk management"  S3 TI "medica* error*" OR AB "medica* error*"  S2 TI safe* OR AB safe*  S1 TI "patient safety" OR AB "patient safety" | 197 | 15 | 0 |

# **OAIster**

| **#** | **Search terms** | | | **Total search yield** | **Potentially relevant records / full text review** | **Included** |
| --- | --- | --- | --- | --- | --- | --- |
| 1 | “Measuring safety” | AND | "Ireland" | n=2 | n=0 | n=0 |
| 2 |  |  | "Irish hospitals" | n=0 | n=0 | n=0 |
| 3 |  |  | "Irish healthcare" | n=0 | n=0 | n=0 |
| 4 | “Monitoring safety” | AND | "Ireland" | n=0 | n=0 | n=0 |
| 5 |  |  | "Irish hospitals" | n=0 | n=0 | n=0 |
| 6 |  |  | "Irish healthcare" | n=0 | n=0 | n=0 |
| 7 | “Measurement of safety” | AND | "Ireland" | n=0 | n=0 | n=0 |
| 8 |  |  | "Irish hospitals" | n=0 | n=0 | n=0 |
| 9 |  |  | "Irish healthcare" | n=0 | n=0 | n=0 |
| Total= | | | | N=2 | N=0 | N=0 |

# **WHO IRIS**

| **#** | **Search terms** | **Total search yield** | **Potentially relevant records / full text review** | **Included** |
| --- | --- | --- | --- | --- |
| 1 | Title contains: Measuring safety | 17 | 2 | 0 |
| 2 | Title contains: Monitoring safety | 12 | 0 | 0 |
| 3 | Title contains: Measurement of safety | 17 | 2 | 0 |

# **Google scholar**

| **#** | **Search terms** | | **Total search yield** | **Potentially relevant records / full text review** | **Included** |
| --- | --- | --- | --- | --- | --- |
| 1 | “Measuring safety” | "Ireland" | n= first 100 hits | n=1 |  |
| 2 |  | "Irish hospitals" | n=8 | n=1 |  |
| 3 |  | "Irish healthcare" | n=9 | n=1 |  |
| 4 | “Monitoring safety” | "Ireland" | n= first 100 hits | n=2 |  |
| 5 |  | "Irish hospitals" | n=4 | n=0 |  |
| 6 |  | "Irish healthcare" | n=2 | n=0 |  |
| 7 | “Measurement of safety” | "Ireland" | n=0 | n=0 |  |
| 8 |  | "Irish hospitals" | n=4 | n=0 |  |
| 9 |  | "Irish healthcare" | n=4 | n=0 |  |
| total | | | N=231 | N=5 | N=0 |

**Lenus (the Irish health research repository)**

| **#** | **Search terms** | **Total search yield** | **Potentially relevant records / full text review** | **Included** |
| --- | --- | --- | --- | --- |
| 1 | (Measuring safety [Title/Abstract]) | 97 | 0 | 0 |
| 2 | (Monitoring safety [Title/Abstract]) | 60 | 3 | 0 |
| 3 | (Measurement of safety [Title/Abstract]) | 97 | 0 | 0 |

**HSE**

| **#** | **Search terms** | **Total search yield** | **Potentially relevant records / full text review** | **Included** |
| --- | --- | --- | --- | --- |
| 1 | Measuring safety | n= first 100 hits | 6 | 0 |
| 2 | Monitoring safety | n= first 100 hits | 1 | 0 |
| 3 | Measurement of safety | n= first 100 hits | 3 | 1 |

**Department of Health**

| **#** | **Search terms** | **Total search yield** | **Potentially relevant records / full text review** | **Included** |
| --- | --- | --- | --- | --- |
| 1 | Measuring safety | 9 | 0 | 0 |
| 2 | Monitoring safety | 4 | 0 | 0 |
| 3 | Measurement of safety | 9 | 0 | 0 |

**HIQA**

| **#** | **Search terms** | **Total search yield** | **Potentially relevant records / full text review** | **Included** |
| --- | --- | --- | --- | --- |
| 1 | Measuring safety | 22 | 7 | 0 |
| 2 | Monitoring safety | 39 | 2 | 0 |
| 3 | Measurement of safety | 317 | 3 | 0 |
