# Supplementary material for: Measuring and monitoring patient safety in hospitals in the Republic of Ireland
Source: Ir J Med Sci. 2023 Mar 22;192(6):2581–93. doi: 10.1007/s11845-023-03336-3 (PMC10692269; doi:10.1007/s11845-023-03336-3)
Supplement: Supplementary file 4 — Supplementary file4 (DOCX 70 KB) [file 11845_2023_3336_MOESM4_ESM.docx]

**Additional File 2**

**Document analysis**

1. Harm- have we been safe in the past?

2. Reliability of safety critical processes

3. Sensitivity to operations- are we safe today?

4. Anticipation and preparedness- will we be safe in the future?

5. Integration and learning- are we responding and improving?

| **Measure** | **Dimension** |
| --- | --- |
| **1^st^ document: Patient Safety Strategy 2019-2024** | |
| P6. a National Clinical Guideline for a National Early Warning Score (NEWS) | 2 |
| P6. Annual National Patient Experience Survey. | 4 |
| P8. working with and learning from patients to design, deliver, evaluate and improve care. | 3 |
| P8. embed a culture of learning and improvement that is compassionate, just, fair and open | 4 |
| P8. increased emphasis on proactively identifying risks | 4 |
| P8. reduce patient harm, with particular focus on the most common causes of harm. | 1 |
| P8.use information from various sources to provide intelligence that will help us recognise when things go wrong, learn from and support good practice and measure, monitor and recognise improvements in patient safety | 5 |
| P8. embed a culture of patient safety improvement through effective leadership and governance | 4 |
| P8. patients are supported with the knowledge, skills and supports that they need to take responsibility for improving their own safety in partnership with staff. | 3 |
| P8. patients, families, carers and advocates are listened to and actively involved in making our services safer. | 3 |
| P11. patients are fully informed and engaged in decisions about their care and are facilitated to best support their own safety. | 3 |
| P11. empower patients to contribute to the safety of health and social care services. This will include their involvement as partners in key governance structures and processes. | 3 |
| P11. National Care Experience Programme to listen to and act on the voice of patients. | 3 |
| P11. training and information needs of patients, families, carers, patient representatives and advocates to enable them to contribute to preventing harm and improving patient safety | 3 |
| P11. embed a culture where we acknowledge when things go wrong, offer meaningful apologies, and act to put things right | 5 |
| P.13 assess, plan and manage workforce and resource requirements, using risk based prioritisation, to ensure safe systems of work and safe staffing levels that support improvements to patient safety. | 4 |
| P.13 ensure that staff are effectively listened to, communicated with and are fully involved and engaged in the planning and delivery of the services they provide and that they are supported and facilitated to raise safety concerns and improve patient safety. | 3 |
| P.13 enhance the capacity and capability of health and social care services and staff to improve patient safety by designing and delivering safety information and training to include patient safety and reliability science, systems thinking, audit, quality improvement methodologies, change management, human factors and multidisciplinary team working for safety. | 5 |
| P.13 promote behaviours that support a culture of safety, this will include strategies that enhance situational awareness, for example ‘safety pauses’ for teams. | 3 |
| P.13 coordination, networking, sharing and learning for patient safety amongst patient safety leaders, staff, health care providers and external agencies | 5 |
| P.13 support staff in reporting and learning from incidents and implement strategies to enhance and improve incident reporting and reviews. | 1,5 |
| P.13 measure the culture of patient safety across health and social care services and identify and implement actions to address identified deficits. | 4 |
| P15. Key strategic and policy decisions taken by management teams will be routinely risk assessed so that unintended consequences that might impact on patient safety are avoided. | 4 |
| P15. systems to continuously improve the quality and analysis of patient safety data and intelligence to allow us assess risks to patient safety | 4 |
| P15. quality and timeliness of incident reviews and ensure that learning from the review of incidents is optimised | 5 |
| P15. formal processes for the communication of risk in line with the organisation’s accountability arrangements. | 5 |
| P15. integrate patient safety information and data to allow us to analyse the reliability of health and social care processes | 2 |
| P15. proactively identify areas of risk to patient safety, | 4 |
| P15. and learn from where things go wrong and from examples of good practice in a way that will inform safety improvement programmes. | 5 |
| P15. publish data in relation to patient safety across the health and social care system. | 5 |
| P15. put in place resourcing for the full implementation of National Clinical Guidelines | 4 |
| P15. strengthen clinical audit structures and processes | 2 |
| P17. develop implementation plans and prioritise initiatives to address these and other emerging priorities for patient safety improvement as part of our annual and multi annual planning process over the course of the Strategy’s lifetime | 5 |
| P17. constantly monitor and review patient safety risks and will prioritise other patient safety and improvement initiatives where this is required. | 4 |
| P19. develop and enhance local and national suites of key patient safety indicators | 1 |
| P19. develop, consolidate and continuously improve patient safety surveillance and reporting systems at every level of the health and social care service. | 1 |
| P19. use a range of information sources and methods of presenting data, including incident and risk data, quality and safety metrics for clinical service, | 5 |
| P19. assessments against national standards, | 1 |
| P19. patient engagement, staff engagement, | 3 |
| P19. claims, | 1 |
| P19. complaints, | 1 |
| P19. incident reviews, | 1 |
| P19. clinical audit, | 1 |
| P19. regulatory reports, | 1 |
| P19. mortality reviews, | 1 |
| P19. and research to support these patient safety surveillance and reporting systems. | 5 |
| P19. publish reports in relation to our performance in patient safety and we will recognise and highlight achievements in patient safety improvement. | 5 |
| P19. measure compliance with the National Standards for Safer, Better Healthcare | 1 |
| P19. support patient safety research and publish and act on the results | 5 |
| P19. develop and enhance technology solutions, including eHealth, to improve access to and reliability of information to measure and improve patient safety. | 5 |
| P21. align staff skilled in quality and patient safety with patient safety initiatives | 4 |
| P21. comprehensive communications programme and supporting awareness campaign to engage support for patient safety amongst the public and health and social care staff and to disseminate learning and good practices. | 5 |
| **2^nd^ Document: Building a Culture of Patient Safety: Report of the Commission on Patient Safety and Quality Assurance** | |
| P17. A national network of patient advocates who will work in partnership with healthcare organisations and other key players to improve patient safety should be identified, supported and developed through appropriate training programmes; the network should also, where appropriate, have strong links with international/worldwide initiatives. | 3 |
| P18. Provision should be made for patient and family involvement in research activities such as measuring patient contribution to bad outcomes, factors that rescue patients from provider error, and factors that mitigate the harm caused by errors. | 5 |
| P52. A confidential web-based national clinical incident reporting system, STARSWeb | 1 |
| P52. A very important objective for Clinical Indemnity Scheme (CIS) is sharing of learning to support patient safety. This is done in a variety of ways: as claims are closed, they are subjected to analysis in order to capture any learning from them. Feedback is provided to the individual enterprise and any generic lessons are fed back into the system through workshops, seminars, the Clinical Indemnity Scheme (CIS) website or newsletter | 5 |
| P56. patient forums and expert patient groups in engagement in service design, planning and evaluation | 3 |
| P.153. Clinical audit | 2 |
| **3^rd^ document: Key Performance Indicator Metadata 2021** | |
| P64. Rate of new cases of hospital acquired Staphylococcus aureus bloodstream infection | 1 |
| P65. Rate of new cases of hospital associated C. difficile infection | 1 |
| P66. % of acute hospitals implementing the requirements for screening of patients with Carbapenemase Producing Enterobacterales (CPE) guidelines | 2 |
| P67. % of acute hospitals implementing the national policy on restricted antimicrobial agents | 2 |
| P69. Rate of medication incidents as reported to NIMS per 1,000 beds | 1 |
| P70. % of hospitals implementing Irish National Early Warning System (INEWS) in all clinical areas of acute hospitals (as per 2019 definition) | 2 |
| P72. % of hospitals implementing The Irish Paediatric Early Warning System (PEWS) | 2 |
| P74. % of hospitals that have completed a self-assessment against all 53 essential elements of the National Standards for Safer, Better Healthcare | 2 |
| P75. % of acute hospitals that have completed and published monthly hospital patient safety indicator reports | 2 |
| P83. % of maternity units / hospitals with full implementation of Irish Maternity Early Warning System (IMEWS) (as per 2019 definition) | 2 |
| P85. % of all hospitals implementing IMEWS (as per 2019 definition) | 2 |
| P87. % of maternity hospitals/units that have completed and published monthly Maternity Safety Statements | 2 |
| P88. % of Hospital Groups that have discussed a quality and safety agenda with NWIHP on a bi/quarterly/monthly basis in line with the frequency stipulated by NWIHP | 2 |
| P116. No. of new cases of CPE (Carbapenemase Producing Enterobacterales) | 1 |
| P117. Rate of venous thromboembolism (VTE, blood clots) associated with hospitalisation | 1 |
| **4^th^ Document: National Standards for the** **Conduct of Reviews of Patient Safety Incidents 2017** |  |
| P6. Standard 1 Service providers support a culture of patient safety that promotes trust, openness, empathy and respect in the review of patient safety incidents. | 4 |
| P21. Standard 1.5 Service providers promote a culture of welcoming feedback, compliments, complaints and concerns in relation to conducting reviews of patient safety incidents. This information is used effectively to improve safety and promote learning throughout the service. | 5 |
| P6. Standard 2 Service providers have formal governance structures in place service-wide for assuring timely and effective reviews of patient safety incidents. | 2 |
| P22. Standard 2.1 Governance structures are in place which ensure the service effectively reviews patient safety incidents, minimises the risk of harm to service users and implements actions and learning from reviews of patient safety incidents. | 2 |
| P22. Standard 2.2 Governance structures promote patient safety as a collective goal within the service to support the timely and effective review of patient safety incidents, including adherence to due process and fair procedure. | 2 |
| P22. Standard 2.3 Service providers have integrated corporate and clinical governance structures which define roles, accountability and responsibilities throughout the service for conducting reviews of patient safety incidents. | 2 |
| P22. Standard 2.4 Service providers demonstrate visible leadership in promoting a just culture of openness, quality and safety in the review of patient safety incidents through: allocation of resources and training | 4 |
| and monitoring and evaluation processes. | 2 |
| P22. Standard 2.5 Service providers have a standardised approach to the conduct of reviews of patient safety incidents service-wide in the following areas: reporting and escalation process, arrangements for feedback, staff skills and experience, workforce planning, including capacity building and protected time, implementation of recommendations from reviews, sharing the learning for improvement. | 2 |
| P23. Standard 2.6 Governance structures are in place to assess service-wide performance and proactively monitor, analyse (including historical and trend analysis) and respond to information relevant to the review of patient safety incidents. This information includes: | 4 |
| audits, including clinical audits | 2 |
| surveys, including experience surveys and patient safety culture surveys | 4 |
| complaints, compliments and concerns | 1 |
| findings from risk assessments | 4 |
| legal claims | 1 |
| findings and recommendations from local, national and international reviews and investigations. | 5 |
| P23. Standard 2.8 Service providers have governance structures in place for positive and cooperative relationships with other agencies, as appropriate, to support the effective review of patient safety incidents; this includes procedures on information sharing and interagency working. | 5 |
| P6. Standard 3 Service providers have clear lines of accountability in place service-wide for the conduct of reviews of patient safety incidents. | 2 |
| P6. Standard 4 Service providers implement a service-wide system to monitor and evaluate the effectiveness of reviews of patient safety incidents. | 2 |
| P25. Standard 4.1 Service providers monitor the conduct of reviews of patient safety incidents on a monthly basis in adherence with relevant national policy, standards and guidelines. | 2 |
| P25. Standard 4.2 Service providers publish an annual overview report on the conduct of reviews of patient safety incidents. This should include adherence to time frames for reviews and how actions and recommendations from reviews are being implemented in the service. | 5 |
| P25. Standard 4.3 Service providers evaluate the systems for monitoring the effectiveness of the conduct of reviews of patient safety incidents on an annual basis. | 2 |
| P25. Standard 4.5 Service providers evaluate the findings of reviews of patient safety incidents and any actions required and share relevant learning locally and nationally to improve the quality and safety of the service. | 5 |
| P25. Standard 4.6 Service providers evaluate the incident review process and incident review reports to identify opportunities for improvement for implementation service- wide. | 5 |
| P25. Standard 4.7 Service providers, in consultation with service users and staff, develop and implement quality improvement programmes to actively improve services based on the learning from reviews of patient safety incidents. These programmes are evaluated annually. | 5 |
| P6. Standard 5 Service providers have effective information governance structures in place service-wide for the management of information related to reviews of patient safety incidents. | 2 |
| P26 Standard 5.5 There is an annual evaluation of the service’s record management practices and systems for information related to the review of patient safety incidents. | 2 |
| P6. Standard 6 Service users and their families are actively engaged with as part of the review of patient safety incidents, and their views are listened to, respected and responded to in a timely manner. | 3 |
| P29. Standard 6.4 Service users and their families are facilitated to provide feedback on their experience of the review process. Where areas for improvement are identified, the service provider takes action to address the issues raised. | 5 |
| P6. Standard 7 Service users and families involved in a patient safety incident are appointed a service-user liaison to facilitate communication with the incident management/review team and access to support. | 3 |
| P7. Standard 10 Service providers establish a standing incident management team to oversee the management and review of patient safety incidents. | 2 |
| P7. Standard 15 Service providers ensure a preliminary assessment of the patient safety incident takes place, and the decision on the appropriate level of review required is clearly documented. | 2 |
| P7. Standard 17 Reviews of patient safety incidents are conducted in a timely manner, in line with the service’s policy and procedures. | 2 |
| P7. Standard 19 Service providers implement the recommendations and actions from patient safety incident-review reports. | 5 |
| P8: Standard 20 Service providers have structures in place to actively share the learning from reviews of patient safety incidents service-wide. | 5 |
| P.48 Standard 20.3 Service providers actively promote discussion on the learning from reviews of patient safety incidents to promote a positive safety culture service-wide. | 3 |
| P48. Standard 20.6 Service providers work in partnership with external bodies, as appropriate, to share the learning from reviews of patient safety incidents. | 5 |
| **5^th^ Document: The Incident Management Framework 2020** |  |
| P17. identifying areas where incidents are likely to occur and putting in place systems to prevent or reduce the likelihood of the risk of their occurrence. | 4 |
| P17. service’s risk management and quality improvement processes are informed by information from a variety of sources such as but not limited to incidents, complaints, claims management, coroner’s reports, and regulatory inspection | 5 |
| P17. The Quality and Safety Committee or equivalent has a key role in promoting, monitoring and sharing learning from the services’ quality and safety processes. | 5 |
| P20. It is the responsibility of the staff member identifying the incident to report the incident either by completion of the appropriate National Incident Report Form (NIRF) or direct entry to NIMS if available | 1 |
| P20. All maternal deaths must also be reported to the NWIHP within 48 hours of occurrence. | 1 |
| P22. Category 1 incidents must be referred to the Serious Incident Management Team (SIMT) | 2 |
| P23. Systems Analysis: A structured process that aims to identify what happened, how and why it happened, what can be done to reduce the risk of recurrence and make services safer. | 5 |
| P23. After Action Review (AAR): This is a structured facilitated discussion of an event, the outcome of which enables the individuals involved in the event to understand why the outcome differed from that which was expected and what learning can be identified to assist improvement | 5 |
| P28. Systems for monitoring the progression of reviews should be established to ensure that it is completed within timeframes | 2 |
| P31. Sharing Learning from the Review Report : At a minimum, services must arrange to have final reports discussed at the relevant Committee, for example, the Quality and Safety Committee. This is to ensure that any learning identified can be shared internal to the service for the purpose of patient safety and quality improvement. | 5 |
| P31. Consideration should also be given to the completion of a learning summary which sets out a brief description of the background to the incident and the learning adduced. Such summaries can then be shared with services beyond the particular service/organisation within which the incident occurred | 5 |
| P31. Publication of the Review Report: Publication in this regard means putting the report in the public domain. Reports relating to service user incidents are personal to the service user and their relevant person(s) and as such are not generally published. If there is a request for such a report to be published, this request should be discussed with the Hospital Group CEO, CHO CO or equivalent senior manager. | 5 |
| P32. Look Back Reviews: A Look Back Review is a process that is initiated where it has been determined that a number of people have been exposed to a specific hazard. The process seeks to identify if any of those exposed to the hazard have been harmed and what needs to be done to ameliorate the harm. | 1 |
| P33. Cross service reviews: Due to the manner in which care is delivered, incidents may cross organisational/care boundaries, for example, pre-hospital/hospital care, hospital/community care, inter-hospital transfers, mental health/acute care, etc. | 1 |
| P33. external reviews/investigations e.g. An Garda Síochána | 1 |
| P33. Reviews relating to Multi-Incident Events: Multi-incident events may arise where a cluster of similar incidents are identified by the service or concerns are received by a service from members of the public relating to an aspect of service provision. Clusters of concerns may in particular arise in the aftermath of the public reporting of an incident(s). | 1 |
| P35. It is recommended that rather than monitor action plans for individual reviews, that action plans developed are interfaced with the relevant service improvement plan with implementation monitored via these. | 5 |
| P35. In cases where monitoring of implementation is being done through the service improvement plan, a record should be kept of the actions in the service improvement plan which relate to the implementation of recommendations in a particular review report | 2 |
| P35. A monitoring process must be in place to track the completion of actions and where there is evidence that actions are behind schedule, appropriate corrective action must be taken to address this. | 2 |
| P35. At a minimum, progress on implementation of actions from Category 1 incidents must be reported to the relevant committee e.g. Quality and Safety Committee on a quarterly basis. | 5 |
| P35. Reports relating to aggregate analysis of data from NIMS and thematic learning should be collated over specific timeframes to assist and inform the wider service improvement programmes. | 5 |
| P35. Services should have in place systems to verify implementation and monitor the effectiveness of improvement strategies which aim to improve the safety and quality of services. The use of audit and monitoring should be central to this and should occur both at the unit level and organisational level through the relevant committee e.g. Quality and Safety Committee. | 2 |
| P35. Services are required to publish an annual overview report in relation to incident reporting and management. This should include detail of incidents reported by type, speciality, and severity, compliance with key performance indicators relating to incident management and detail on how the actions and recommendations from reviews are being implemented in the service to improve safety. | 5 |
| **6^th^ documents: National Standards for Safer Better Healthcare** |  |
| P20. Standard 1.1.1 Proactive and systematic identification of service users’ collective needs and preferences. | 4 |
| P20. Standard 1.1.7 Feedback from service users being used to continuously improve the experience for all service users. | 5 |
| P34. Standard 1.8.3 Complaints procedures that identify the expectations of service users making complaints and ensure that these expectations are taken into account and addressed throughout the process. | 5 |
| P42. Standard 2.1.2 Use of National Clinical Guidelines and nationally agreed protocols, care bundles and care pathways where available. | 2 |
| P42. Standard 2.1.3 Regular reviews of National Clinical Guidelines to determine what is relevant to the care and treatment provided and taking steps to address any identified gaps to ensure guidelines are implemented. | 1 |
| P42. Standard 2.1.4 A clearly documented risk assessment when services are unable to fully implement National Clinical Guidelines and appropriate action taken to ensure the quality and safety of services. | 4 |
| P52. Standard 2.6.4 Regular review of the services provided and evidence that the defined model of service can be delivered safely. | 2 |
| P53. Standard 2.6.5 Ongoing assessment of the volumes and casemix of their service users to ensure services are provided to sufficient numbers of service users to maintain the skills and competencies of clinical teams based on best available evidence or advice from the relevant professional and expert bodies. | 4 |
| P53. Standard 2.6.6 Management of available resources, including the workforce, to meet legislative requirements, and to deliver the defined model of service safely and sustainably at all times. | 4 |
| P54. Standard 2.7.9 The proactive identification of risks associated with changes to the physical environment where care is delivered and evaluation of identified risks and necessary action to eliminate or minimise such risks. | 4 |
| P56. Standard 2.8.1 Use of relevant national performance indicators and benchmarks, where they exist, to monitor and evaluate the quality and safety of the care and its outcomes. | 1 |
| P56. 2.8.5 Monitoring and evaluation of performance by developing and implementing clinical and non-clinical audits | 1 |
| and implementing improvements based on the findings. | 5 |
| P56. 2.8.8 Clinical governance arrangements that ensure findings from clinical audits are reported and monitored effectively. | 5 |
| P62. 3.1.1 Proactive monitoring, analysis and response to information relevant to the provision of safe services. This information includes: patient-safety incidents complaints, concerns and compliments, findings from risk assessments, legal claims, audits, satisfaction surveys, findings and recommendations from national and international reviews and investigations, casemix, activity and performance data. | 4 |
| P64. Standard 3.2 Service providers monitor and learn from information relevant to the provision of safe services and actively promote learning both internally and externally. | 5 |
| P66. Standard 3.3 Service providers effectively identify, manage, respond to and report on patient-safety incidents. | 1 |
| P66. Standard 3.3.7 Evaluation of the effectiveness of the arrangements for identifying, managing, responding to and reporting on patient-safety incidents. | 2 |
| P72. 3.6.2 Clear articulation of the elements of a patient-safety culture and specific arrangements that actively promote this culture through a mission statement, service design, code of conduct, allocation of resources and training, development and evaluation processes. | 4 |
| P74. 3.7.2 A patient-safety improvement programme based on assessed local needs and priorities and national and international initiatives. This programme incorporates specific evidence-based interventions that are proportionate to the context, nature and scale of the service provided. | 5 |
| P86. Standard 5.1 Service providers have clear accountability arrangements to achieve the delivery of high quality, safe and reliable healthcare. | 2 |
| P88. Standard 5.2 Service providers have formalised governance arrangements for assuring the delivery of high quality, safe and reliable healthcare. | 2 |
| P96. 5.6.4 Regular review and identification of areas for improvement in the culture of the service, which incorporates feedback from service users and the workforce. | 5 |
| P100. 5.8.7 Proactive approach to learning from findings and recommendations from national and international reviews and investigations. | 5 |
| P112. Standard 6.1 Service providers plan, organise and manage their workforce to achieve the service objectives for high quality, safe and reliable healthcare. | 4 |
| P116. Standard 6.3.5 A training, educational and development programme with a specific focus on patient safety, communication and person-centred care, which has clear objectives and which is tailored to specific members of the workforce to develop competencies in order to ensure the delivery of high quality safe care. | 5 |
| P116. 6.4.5 Monitoring, management and development of the performance of the workforce, at individual and team level, including the evaluation of service users’ feedback and taking action to address identified areas for improvement. | 5 |
| P124. 7.1.3 Consultation with key stakeholders including service users, policy makers and their workforce regarding the allocation of resources to achieve the best quality and safety outcomes for service users. | 4 |
| P126. 7.2.2 Regular evaluation and management of the efficiency and cost- effectiveness of services and technologies. This evaluation and management uses best available evidence to maximise quality and safety and to inform investment and disinvestment decisions. | 4 |
